# Epithelial-Mesenchymal Transition Gene Signature Is Associated with Neoadjuvant Chemoradiotherapy Resistance and Prognosis of Esophageal Squamous Cell Carcinoma

**DOI:** 10.1155/2022/3534433

**Published:** 2022-08-27

**Authors:** Kewei Song, Baohong Gu, Chenhui Ma, Bofang Wang, Na Wang, Rong Yu, Hao Chen

**Affiliations:** ^1^The Second Clinical Medical College, Lanzhou University, Lanzhou 730030, China; ^2^Department of Tumor Surgery, Lanzhou University Second Hospital, Lanzhou 730030, China; ^3^Key Laboratory of Digestive System Tumors of Gansu Province, Lanzhou 730030, China

## Abstract

**Background:**

Neoadjuvant chemoradiotherapy (neo-CRT) in combination with surgery increases survival compared to surgery alone, as indicated by the esophageal squamous cell carcinoma (ESCC) treatment recommendations. However, the benefits of neo-CRT are diverse among patients. Consequently, the development of new biomarkers that correlate with neo-CRT might be important for the treatment of ESCC.

**Methods:**

The differentially expressed genes (DEG) between responsive and resistant samples from the GSE45670 dataset were obtained. On the TCGA dataset, survival analysis was performed to identify prognosis-related-EMT-genes. For EMT score model construction, lasso regression analysis in the TCGA cohort was used to identify the genes. In the TCGA-ESCC cohort, age, stage, and EMT score were used to construct a nomogram.

**Results:**

In total, 10 prognosis-related-EMT-genes were obtained. These 10 genes consisted of 6 risky genes and 4 protective genes. Based on the lasso analysis and univariate Cox regression, an EMT score model consisting of 7 genes (CLEC18A, PIR, KCNN4, MST1R, CAPG, ALDH5A1, and COX7B) was identified. ESCC patients with a high EMT score have a worse prognosis. These genes were differentially expressed between responsive and resistant patients and had a high accuracy for distinguishing resistant and responsive patients.

**Conclusions:**

The identified genes have the potential to function as molecular biomarkers for predicting ESCC patients' resistance to neo-CRT. This research may aid in the elucidation of the molecular processes driving resistance and the identification of targets for improving the prognosis for ESCC.

## 1. Introduction

There will be 20,640 new cases of esophageal cancer diagnosed in the United States in 2022, and 16410 people will die from esophageal cancer, according to the 2022 Cancer Statistics for the United States [1]. ESCC, a major histological subtype of EC, accounts for roughly 90% of EC occurrences [2, 3]. A number of factors contribute towards the development of ESCC, including smoking, alcohol abuse, and hot water [4, 5]. ECSS can be difficult to diagnose because there are no specific symptoms and a lack of early detection methods that allow an early diagnosis [6]. Only 15-25% of patients with ESCC survive five years after they were initially diagnosed with the disease [7]. To increase the survival time of ESCC, it is urgently necessary to discover the genetic changes of ESCC and identify new biomarkers.

The most common treatment for locally advanced ESCCs is surgery [7]. It is important to note that disease recurrences are common after surgery, and that the prognosis has not changed significantly over the past few decades [8]. The use of neo-CRT in conjunction with surgery improves prognosis greatly as compared to surgery alone, and it is suggested in treatment recommendations [9]. In a trial including 113 patients with esophageal cancer, the addition of neo-CRT increased the 3-year survival rate from 6% to 32% [10]. It should be mentioned, however, that neo-CRT had two major disadvantages. Initially, the outcomes of neo-CRT treatment are variable. Some patients could be resistant to neo-CRT and have a worse prognosis in terms of survival [11]. In addition, studies have revealed that neo-CRT is linked with an increased risk of postoperative complications [12]. Therefore, it would be beneficial to ESCC patients if novel biomarkers could be identified that would predict their response to neo-CRT.

It is quite common for cancer cells to activate diverse signaling pathways and develop chemotherapy resistance, which helps them stay alive in spite of chemotherapy [13]. In studies, it has been observed that chemotherapy resistance can be caused by the epithelial–mesenchymal transition (EMT) [14–16]. EMT is generally defined as the loss of epithelial characteristics in a cell and the acquisition of mesenchymal characteristics in that same cell [17]. There is increasing evidence that EMT is linked to tumorigenesis, cancer invasion, and drug resistance [18, 19]. It has been found that there is a significant association between EMT genes and metastatic disease, as well as the clinical stage of ESCC [20]. However, more studies are needed to investigate the role of EMT in ESCC.

The expression data of responsive and resistant samples was obtained from different databases. The differentially expressed EMT-related genes that are correlated with neo-CRT responsiveness were identified. Using lasso regression analysis, 7 genes (CLEC18A, PIR, KCNN4, MST1R, CAPG, ALDH5A1, and COX7B) were used to obtain the EMT score for estimating the ESCC prognosis. Besides, EMT score, age, and stage were used for the construction of a nomogram for predicting the 1-, 3-, and 5-year overall survival (OS) of ESCC. For diagnosis (resistant and responsive), the EMT score showed a more accurate value than genes.

## 2. Patients and Methods

### 2.1. Gene Expression Data

GSE45670 expression data was downloaded from the GEO [21] by the GEOquery package [22]. This dataset consisted of 28 esophageal squamous cell carcinoma (ESCC) samples and 10 normal samples. In those 28 patients who had ESCCs, neoadjuvant chemoradiation therapy (neo-CRT) that included cisplatin and vinorelbine was given. 11 of them responded completely to the therapy, while 17 others were resistant to treatment. Aside from that, the TCGAbiolinks package was used to download expression information and clinical records of 185 ESCC patients from the TCGA database [23]. GSE86099 used paclitaxel resistant cells and used mRNA transcription files to identify the crucial genes for developing paclitaxel resistance [24]. The detailed information of samples from GSE45670 and TCGA-ESCC is shown in Supplementary Table 1 and Supplementary Table 2.

### 2.2. DEG Identification and Enrichment Analysis

In order to more clearly illustrate the distribution of 11 responsive and 17 resistant samples, a principal component analysis was applied. In order to increase the quality of samples and the number of DEG, the low-quality samples were then removed by the PCA results. We used the edgeR package to detect DEG between responsive and resistant samples based on log2foldchange (FC) > 0.5 and *p* value < 0.05 as cutoff criteria [25]. The enrichment analysis was conducted using the R package “clusterProfiler” [26]. The *p* value < 0.05 was used to distinguish significantly enriched terms.

### 2.3. Survival-Related EMT Gene Identification

A total of 3600 EMT-related genes were retrieved from EMTome [27]. We determined the genes that were substantially linked with prognosis by samples from TCGA-ESCC. Among these survival-related genes, genes with “Coef > 0” were defined as risky genes, and genes with “Coef < 0” were defined as protective genes. A Venn diagram was used to show the overlap of DEGs, EMT genes, and survival-related genes. The overlapped genes were selected as the survival-related EMT genes.

### 2.4. Construction of EMT Score Model

We have determined the candidate prognostic genes by applying lasso regression analysis in the TCGA-ESCC cohort by using the glmnet package [28]. We then used univariate Cox regression analysis to calculate the coefficients for each gene. The mRNA expression and the coefficients associated with these genes were used in the calculation of the EMT score. ESCC patients from the TCGA dataset were divided into low and high subgroups based on the median value. The prognosis difference between low and high groups was compared, and the prognosis prediction ability of the EMT score was calculated.

### 2.5. Development of Nomogram

The TCGA-ESCC cohort included data on age, stage, and EMT score, which were used to construct a nomogram. Calibration curves were generated so that the concordance between the actual survival rate, and the anticipated survival rate could be evaluated. Additionally, the concordance index (C-index) was calculated to assess the capacity of models to forecast prognosis. These analyses were conducted by the package rms.

### 2.6. Immune Score and Immune Cell Infiltration Analyses

By expression profiles, the immune score and the stromal score were calculated using the “estimate” package [29]. By package GSVA [30], the infiltration levels of immune cell populations were determined.

### 2.7. Diagnostic Ability in the Classification of Resistant and Responsive Patients

In this study, we used the pROC package to estimate the area under curve (AUC) to evaluate the prediction ability of drug response to therapy. Then, we also calculated the AUC values of EMT score and genes in classifying ESCC and normal samples.

## 3. Results

### 3.1. DEG Identification

**Figure 1:**
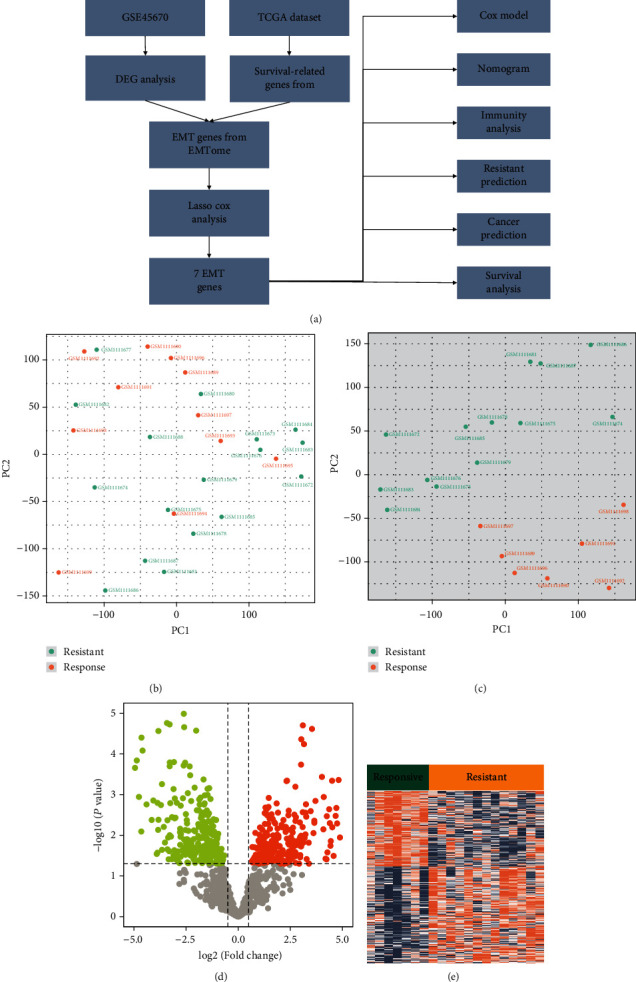
Principal component analysis (PCA) in resistant versus responsive samples. (a) The flowchart of this study. (b) Before removing the outliers, the PCA was performed on the gene expression data. (c) After removing the outliers, the PCA was performed on the gene expression. (d) Volcano plot of DEG by log2 foldchange (FC) > 0.5 and *p* value < 0.05. (e) Clustering heat map of the DEG. The expression data for DEG was normalized.

The flowchart of this study was shown in Figure 1(a). Principal component analysis (PCA) was applied to classify 11 responsive and 17 resistant samples in Figure 1(b). Then, 4 responsive samples (GSM1111699, GSM1111694, GSM1111695, and GSM1111693) and 4 resistant samples (GSM1111677, GSM1111680, GSM1111682, and GSM1111688) were removed since they were outliers (Figure 1(c)). We compared the gene expression between the 7 responsive and 13 resistant samples using the edgeR package. The log2foldchange (FC) > 0.5 and *p* value < 0.05 accepted to consider genes to be differentially expressed, identifying a total of 2604 genes (1142 upregulated and 1462 downregulated in the resistant group) above this cut-off (Figures 1(d) and 1(e)). Then, we investigated the biological processes and pathways by enrichment analysis. External encapsulating structure organization (GO: 0045229), extracellular matrix organization (GO: 0030198), and extracellular structure organization (GO: 0043062) are the main biological processes in DEGs (Table 1). Besides, ECM-receptor interaction (hsa04512), human papillomavirus infection (hsa05165), glycosaminoglycan biosynthesis-chondroitin sulfate dermatan sulfate (hsa00532), and focal adhesion (hsa04510) were the main pathways in DEGs (Table 2).

### 3.2. Survival-Related EMT Gene Identification

**Figure 2:**
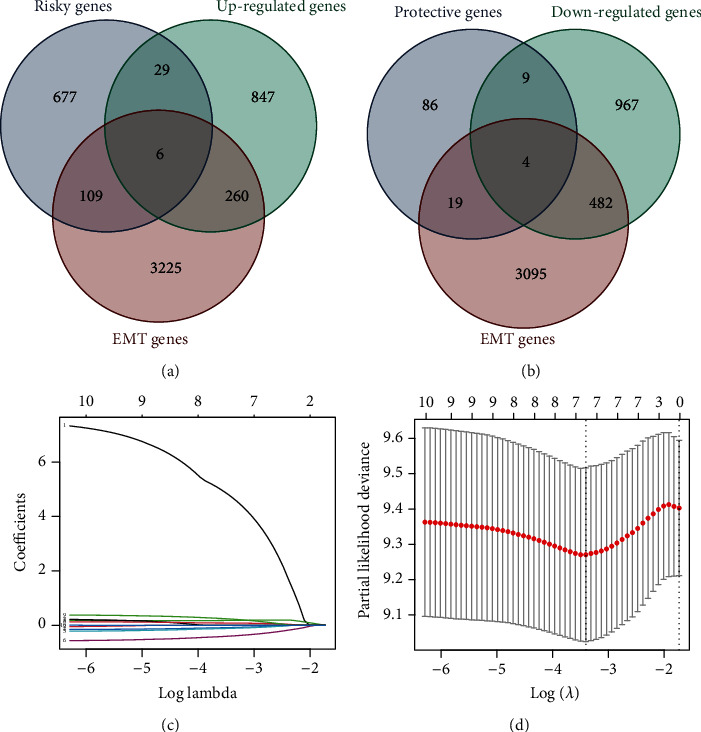
Identification of PREMTs in ESCC. (a, b) Venn diagrams for identifying PREMTs. (c) Lasso coefficient profiles of the 10 PREMTs. (d) Selection of the number of genes for EMT score by lasso analysis.

Based on the survival analysis that was conducted on an R loop, among all 17468 genes, 939 genes were significantly related to survival. Among 939 survival-related genes, 118 were protective genes, and 821 were risky genes. The Venn map shows that 6 EMT genes (PIR, EID3, COX7B, CLEC18A, ALDH5A1, and DYNC1I1) are generally upregulated in resistant samples and are risky genes (Figure 2(a)). Besides, the Venn map shows that 4 EMT genes (CAPG, MST1R, KCNN4, and VDR) are generally downregulated in resistant samples and are protective genes (Figure 2(b)). These ten genes were defined as prognosis-related EMT genes (PREMTs).

### 3.3. Construction of EMT Score

After that, we performed a lasso analysis on the TCGA-ESCC samples to analyze these ten PREMTs (Figure 2(c)). Via the process of cross-validation, it was shown that 7 PREMTs were capable of producing a superior effect in the model (Figure 2(d)). Then, the univariate Cox regression method was adopted to obtain the coefficient values of genes. An EMT model consisting of 7 genes (CLEC18A, PIR, KCNN4, MST1R, CAPG, ALDH5A1, and COX7B) was identified. The EMT score of individuals using coefficients and gene expression was (4.96)∗CLEC18A + (0.36)∗PIR + (−0.18)∗KCNN4 + (−0.24)∗MST1R + (−0.50)∗CAPG + (0.39)∗ALDH5A1 + (0.54)∗COX7B.

**Figure 3:**
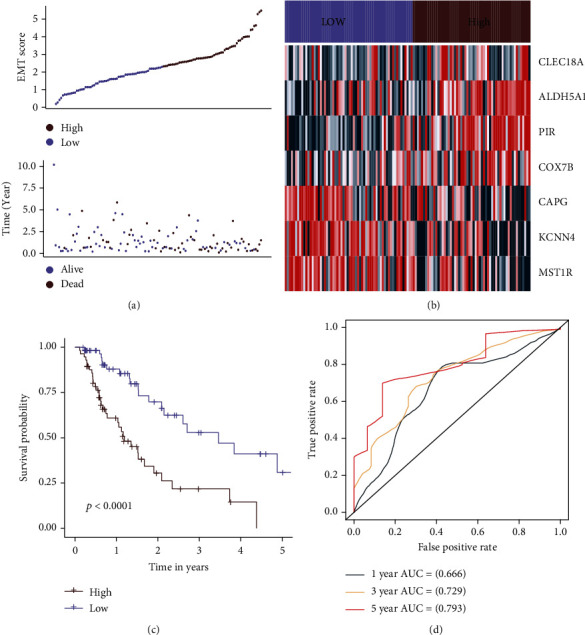
EMT score based on 7 EMT genes. EMT score distribution, survival overview (a), and heat map (b) for patients in the different groups. (c) The survival curves differentiate between groups. (d) The predictive accuracy of the EMT signature for TCGA patients was shown using ROC curves.

Patients with ESCC who were included in the TCGA were classified as having either a high or low EMT score based on the median value. In the course of our research, we examined the rates of mortality in two different EMT groups. We made the startling discovery that the group at high EMT had a survivability that was much lower than the group at low EMT (Figure 3(a)). The expression values of CLEC18A, PIR, KCNN4, MST1R, CAPG, ALDH5A1, and COX7B between groups were illustrated in Figure 3(b). The expression values of CLEC18A, PIR, ALDH5A1, and COX7B were higher in the group at high EMT. The expression values of KCNN4, MST1R, and CAPG were lower in the group at high EMT. There is a substantial difference in OS between groups (*p* value < 0.001, Figure 3(c)). In addition, the AUC value was displayed to assess the EMT signature's predictive abilities. AUC values of the EMT score for 1, 3, and 5 years of survival were 0.662, 0.729, and 0.760, respectively (Figure 3(d)).

### 3.4. The Nomogram for OS Prediction

**Figure 4:**
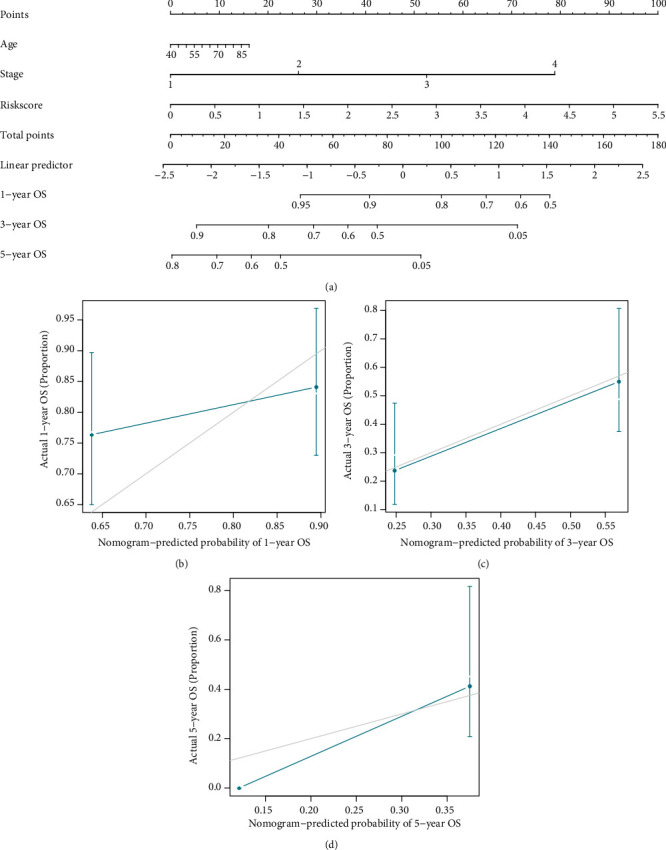
The nomogram constructed in the TCGA-ESCC. (a) The nomogram for predicting OS. The calibration plots for predicting 1-year (b), 3-year (c), and 5-year (d) OS.

Typically, a nomogram is used to quantify the risk of people in a therapeutic environment by combining various variables. By combining the EMT score, age, and stage, we developed a nomogram to estimate the survival rates of 1-, 3-, and 5-year OS of ESCC (Figure 4(a)). Each component in the nomogram is assigned points according to its contribution. The majority of contributions came from the EMT score, and the C-index for the nomogram was 0.70. Calibration curves of 1-, 3-, and 5-years were used to evaluate the accuracy of the model predictions (Figures 4(b)–4(d)). And the findings suggested that actual and anticipated survival were highly concordant, particularly for three-year survival (Figure 4(b)).

### 3.5. Estimation of the EMT Score with Immunity

Using ESTIMATE, the immune and stromal scores were calculated in order to examine the influence of EMT score on tumor immunity. According to the data, the immune score of those with a low EMT score was noticeably higher than those with a high EMT score (*p* value = 0.011, Supplementary Figure 1A). There was an inverse relationship between the EMT score and the tumor immunity (*R* = −0.22, *p* < 0.0001, Supplementary Figure 1B). Patients with a high EMT score, on the other hand, tended to have tumor purity that was greater (Supplementary Figure 1A), but the difference was not significant (*p* value = 0.14).

In addition, the proportions of immune cells were compared across groups (Supplementary Figure 2). The fraction of immune cells such as CD8-T cells, dendritic cells, and natural-killer cells in the low EMT score subgroup was higher than those in the high EMT score subgroup.

### 3.6. Evaluate the Power of Signatures for Distinguishing Resistant and Responsive Patients

**Figure 5:**
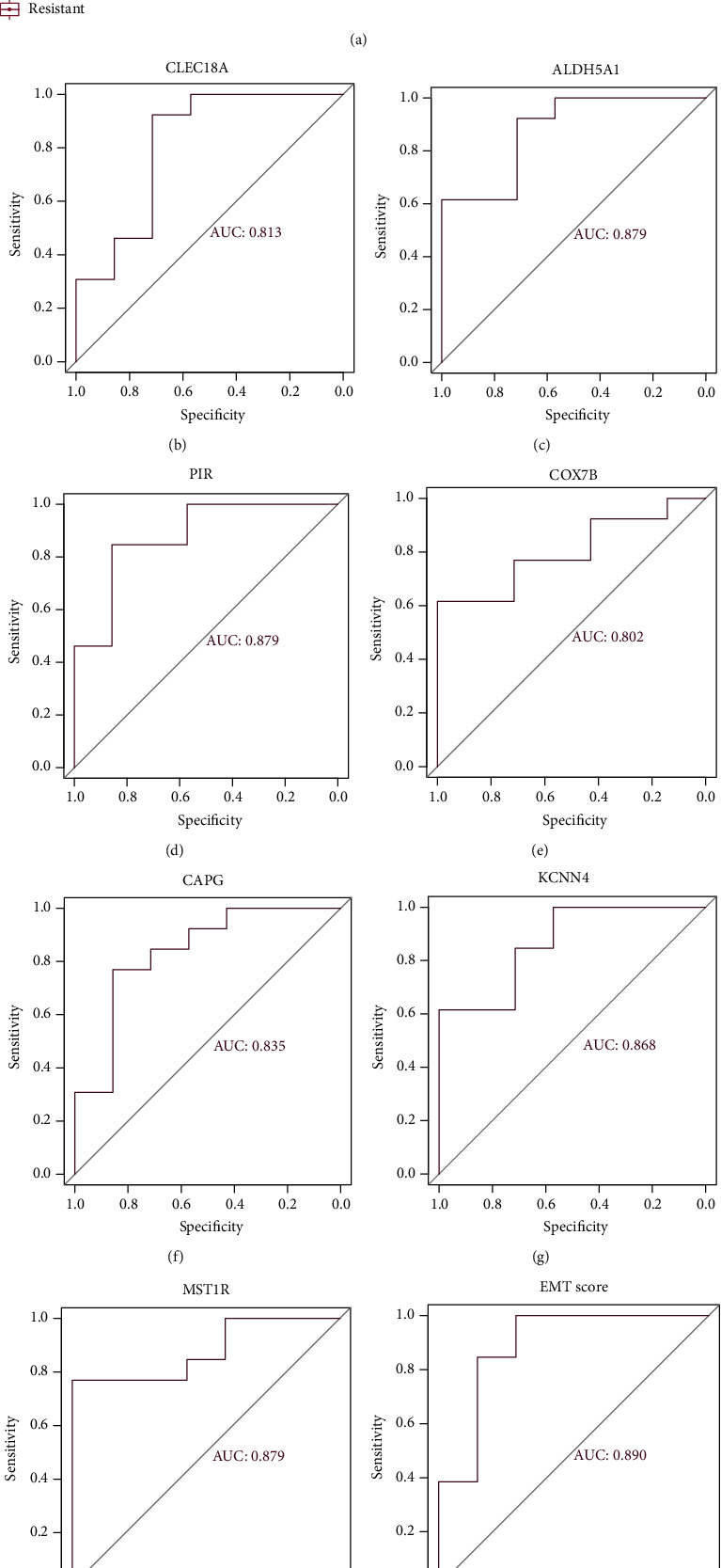
(a) The expression pattern of genes between responsive and resistant patients. ROC curves of genes and EMT score. (b) ROC curve of CLEC18A. (c) ROC curve of ALDH5A1. (d) ROC curve of PIR. (e) ROC curve of COX7B. (f) ROC curve of CAPG. (g) ROC curve of KCNN4. (h) ROC curve of MST1R. (i) ROC curve of EMT score. AUC > 0.7 indicates good effect.

The expression values of genes were compared between resistant and responsive patients (Figure 5(a)). To evaluate the power to distinguish resistant and responsive patients, we measured the AUC of genes and EMT score (Figures 5(b)–5(i)). For diagnosis (resistant and responsive), the EMT score showed the highest AUC value (AUC = 0.89) than genes.

An independent dataset (GSE86099) contains the expression profiles of the cells associated with paclitaxel resistance. For diagnosis (resistant and responsive), all genes and EMT score showed perfect AUC values (AUC = 1.0) (Supplementary Figure 3A–3H).

### 3.7. Evaluate the Power of Signatures for Distinguishing ESCC and Normal Samples

**Figure 6:**
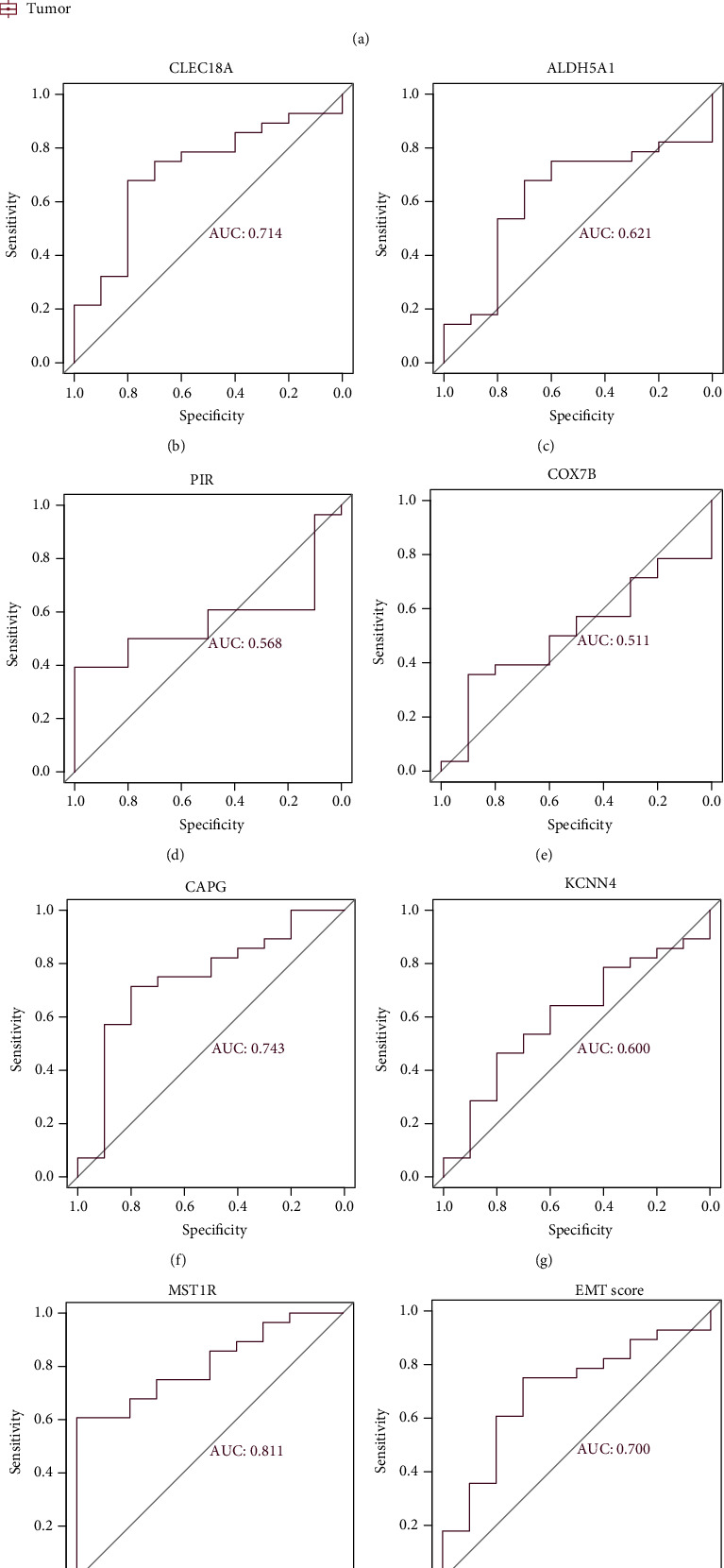
(a) The expression pattern of genes between normal and tumor samples. ROC curves of genes and EMT score. (b) ROC curves of CLEC18A. (c) ROC curves of ALDH5A1. (d) ROC curves of PIR. (e) ROC curves of COX7B. (f) ROC curves of CAPG. (g) ROC curves of KCNN4. (h) ROC curves of MST1R. (i) ROC curves of EMT score. AUC > 0.7 indicates good effect.

The gene expression levels of CAPG, CLEC18A, and MST1R were higher in the tumor samples (Figure 6(a)). We drew the ROC curve of survival-related ECM genes to clarify the diagnostic value for distinguishing ESCC and normal samples (Figures 6(b)–6(i)). The results showed MST1R (AUC = 0.811), CAPG (AUC 0.743), CLEC18A (AUC = 0.714), and EMT score (AUC = 0.700) had significant diagnostic values.

### 3.8. Validates Prognostic Feature Genes

Then, the correlation between EMT genes expression and patient survival was confirmed (Supplementary Figure 4A–4J). The findings demonstrated that patients with elevated levels of ALDH5A1, PIR, and COX7B had a significantly lower OS.

## 4. Discussion

ESCC is a kind of cancer that is aggressive and poses a significant threat to human health as a result of its high incidence rate as well as its low survival rate after 5 years [31]. Currently, there are few effective biomarkers that can be used to diagnose, prognosis, and treatment of ESCC. Expression data was utilized to discover EMT genes linked with chemoradiotherapy resistance, as well as their connection with ESCC prognosis. Finally, 6 risky genes (PIR, EID3, COX7B, CLEC18A, ALDH5A1, and DYNC1I1) and 4 protective genes (CAPG, MST1R, KCNN4, and VDR) were identified. Based on lasso analysis, an EMT score model was constructed by the expression values of 7 genes (CLEC18A, PIR, KCNN4, MST1R, CAPG, ALDH5A1, and COX7B). Patients with an elevated EMT score for ESCC had a worse prognosis.

Earlier research has analyzed the difference in gene expression between nCRT responder and nonresponder samples in order to predict nCRT response [32]. Among the identified genes, five genes could accurately predict the response to nCRT. In our study, among the 7 identified genes, ALDH5A1, CLEC18A, COX7B, and PIR were upregulated in resistant patients. CAPG, KCNN4, and MST1R were upregulated in responsive patients. In the predictive models, all seven genes and EMT score could achieve a high accuracy (>80%) in predicting the response to therapy of patients. Besides, MST1R (AUC = 0.811), CAPG (AUC = 0.743), CLEC18A (AUC = 0.714), and EMT score (AUC = 0.700) also had significant diagnostic accuracy in distinguishing tumor and normal samples.

By analyzing expression profiles, we predicted the immune score and the values of immune subpopulations. According to the findings, the group with the high EMT score had a considerable reduction in the number of immune cells. It is possible that this is the reason why people with high EMT scores have a poorer prognosis. EMT may interact with immunosuppression either directly or indirectly, as shown by the results of a prior study [33]. Since immune cells are important biomarkers for immunotherapy, the influence of EMT on immunity is important and needs more studies.

MST1R was related to cellular motility and matrix invasion that are the predictive indications of a tumor phenotype with the ability to metastasize [34]. MST1R was significantly highly expressed in 74% of gastroesophageal samples, and overexpression predicted poor survival [34]. For other genes, their roles in ESCC need more studies.

There were some limitations to our study. These seven key EMT genes have the potential to be used not only in ESCC resistance prediction but also as possible prognostic biomarkers. However, the association between seven important EMT genes and ESCC prognosis may not be robust. Therefore, in order to discover the precise biological behaviors of these seven genes (CLEC18A, PIR, KCNN4, MST1R, CAPG, ALDH5A1, and COX7B) that are involved in the formation of ESCC, experimental validation has to be carried out. Meanwhile, there were just a few ESCC specimens available. In order to evaluate the potential predictive utility of these genes for illness, more validation in more samples is required.

## 5. Conclusions

Using different datasets, 7 genes that play essential roles in ESCC chemotherapy resistance, namely, CLEC18A, PIR, KCNN4, MST1R, CAPG, ALDH5A1, and COX7B, were selected. The findings of this research may help to clarify the molecular processes of chemotherapy resistance in ESCC and assist us in identifying prospective targets for predicting chemotherapy resistance.

## Figures and Tables

**Table 1:** The GO enrichment analysis results of DEG.

ID	Description	*p* value	Count
GO: 0045229	External encapsulating structure organization	<0.01	118
GO: 0030198	Extracellular matrix organization	<0.01	117
GO: 0043062	Extracellular structure organization	<0.01	117
GO: 0006023	Aminoglycan biosynthetic process	<0.01	40
GO: 0031589	Cell-substrate adhesion	<0.01	86
GO: 0042476	Odontogenesis	<0.01	41
GO: 1903034	Regulation of response to wounding	<0.01	49
GO: 0006024	Glycosaminoglycan biosynthetic process	<0.01	37
GO: 0061041	Regulation of wound healing	<0.01	42
GO: 0001503	Ossification	<0.01	90
GO: 0001667	Ameboidal-type cell migration	<0.01	100
GO: 0050818	Regulation of coagulation	<0.01	27
GO: 0042493	Response to drug	<0.01	81
GO: 0010810	Regulation of cell-substrate adhesion	<0.01	56
GO: 0001501	Skeletal system development	<0.01	101
GO: 0034329	Cell junction assembly	<0.01	91
GO: 0060348	Bone development	<0.01	51
GO: 0022612	Gland morphogenesis	<0.01	35
GO: 0002576	Platelet degranulation	<0.01	38
GO: 0006022	Aminoglycan metabolic process	<0.01	46

**Table 2:** The KEGG enrichment analysis results of DEG.

ID	Description	*p* value	Count
hsa04512	ECM-receptor interaction	<0.01	28
hsa05165	Human papillomavirus infection	<0.01	73
hsa00532	Glycosaminoglycan biosynthesis-chondroitin sulfate dermatan sulfate	<0.01	11
hsa04510	Focal adhesion	<0.01	49
hsa04974	Protein digestion and absorption	<0.01	29
hsa04933	AGE-RAGE signaling pathway in diabetic complications	<0.01	28
hsa05222	Small cell lung cancer	<0.01	26
hsa05146	Amoebiasis	<0.01	27
hsa00480	Glutathione metabolism	<0.01	18
hsa05205	Proteoglycans in cancer	<0.01	45
hsa04151	PI3K-Akt signaling pathway	<0.01	69
hsa04621	NOD-like receptor signaling pathway	<0.01	40
hsa05144	Malaria	<0.01	15
hsa05169	Epstein-Barr virus infection	<0.01	42
hsa00620	Pyruvate metabolism	<0.01	14
hsa05225	Hepatocellular carcinoma	<0.01	36
hsa04360	Axon guidance	<0.01	38
hsa05204	Chemical carcinogenesis - DNA adducts	<0.01	18
hsa04068	FoxO signaling pathway	<0.01	29
hsa05230	Central carbon metabolism in cancer	<0.01	18

## Data Availability

The following information was supplied regarding data availability: data is available at the TCGA (https://portal.gdc.cancer.gov/) and GEO (https://www.ncbi.nlm.nih.gov/geo/).

## Supplementary Materials

Supplementary 1 Supplementary Figure 1: estimation of the EMT score with tumor immunity. (a) Distribution of stromal scores, immune scores, and tumor purity in the low and high EMT score subgroups of the TCGA data cohort. (b) Association between the EMT score and immune score.

Supplementary 2 Supplementary Figure 2: association of the EMT score with the immune subpopulations.

Supplementary 3 Supplementary Figure 3: diagnosis values of (a) CLEC18A, (b) ALDH5A1, (c) PIR, (d) COX7B, (e) CAPG, (f) KCNN4, (g) MST1R, and (h) EMT score for paclitaxel resistance.

Supplementary 4 Supplementary Figure 4: Kaplan–Meier survival curves for genes for ALDH5A1 (a), CAPG (b), CLEC18A (c), COX7B (d), DYNC1I1 (e), EID3 (f), KCNN4 (g), MST1R (h), PIR (i), and VDR (j).

Supplementary 5 Supplementary Table 1: the detailed information of samples from the GSE45670 dataset. Supplementary Table 2: the detailed information of samples from the TCGA-ESCC dataset.
