# Supplementary figures and images for: Epithelial-Mesenchymal Transition Gene Signature Is Associated with Neoadjuvant Chemoradiotherapy Resistance and Prognosis of Esophageal Squamous Cell Carcinoma
Source: Dis Markers. 2022 Aug 27;2022:3534433. doi: 10.1155/2022/3534433 (PMC9442501; doi:10.1155/2022/3534433)

**A**

low high

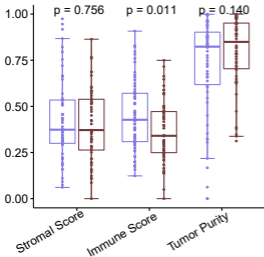**B**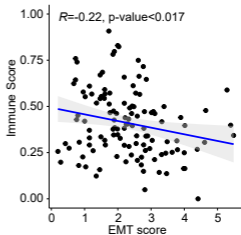

Supplement: Supplementary 1 — Supplementary Figure 1: estimation of the EMT score with tumor immunity. (a) Distribution of stromal scores, immune scores, and tumor purity in the low and high EMT score subgroups of the TCGA data cohort. (b) Association between the EMT score and immune score. [file 3534433.f1.pdf]

low high

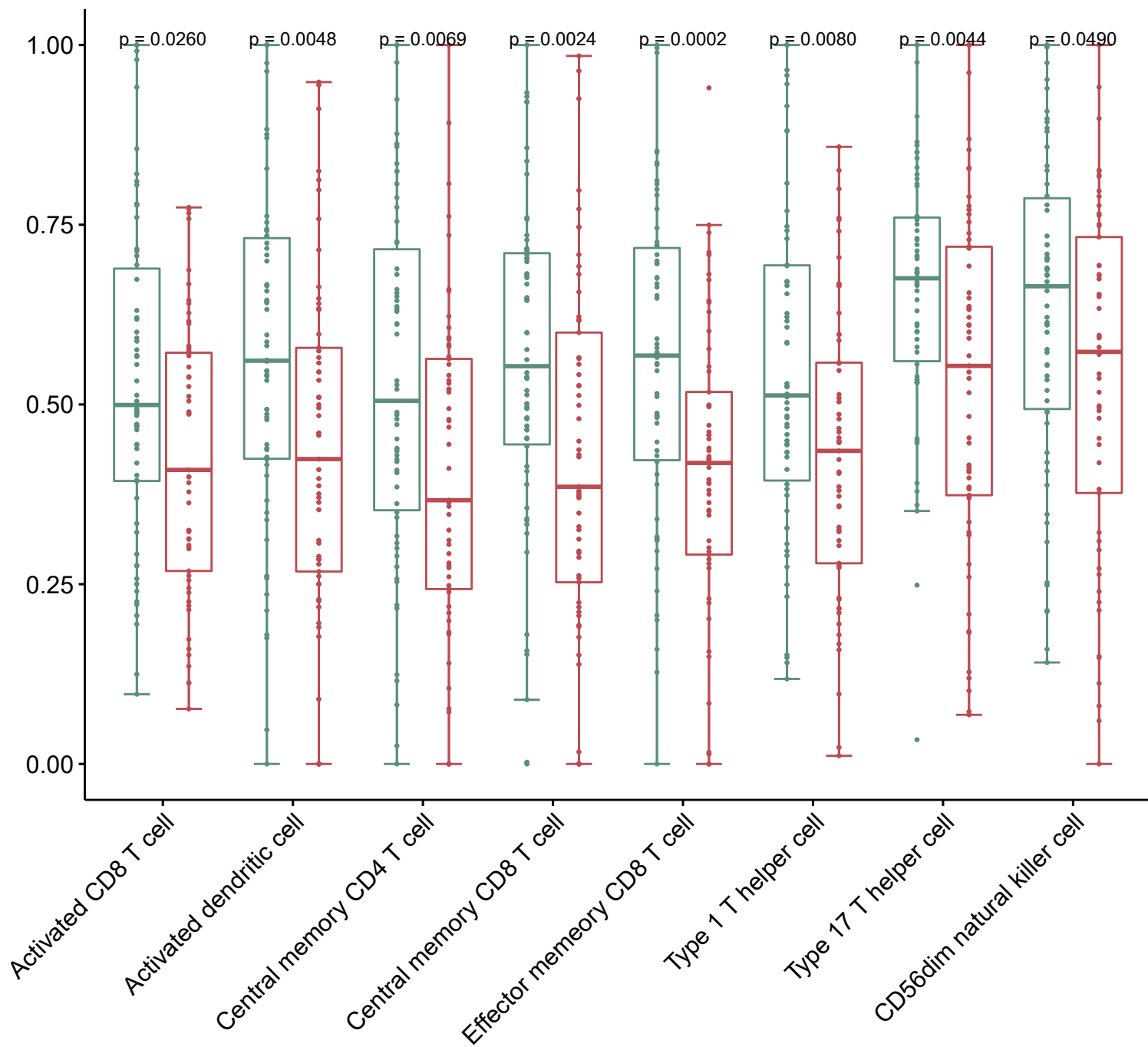

Supplement: Supplementary 2 — Supplementary Figure 2: association of the EMT score with the immune subpopulations. [file 3534433.f2.pdf]

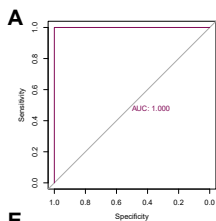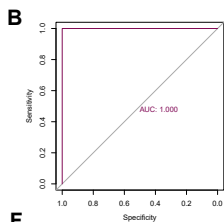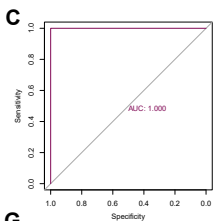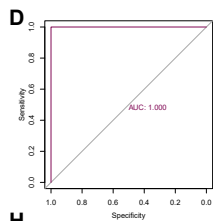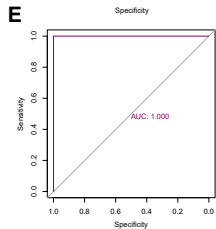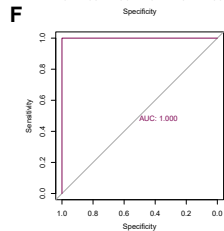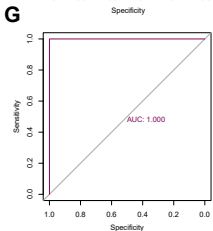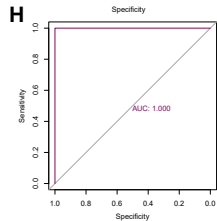

Supplement: Supplementary 3 — Supplementary Figure 3: diagnosis values of (a) CLEC18A, (b) ALDH5A1, (c) PIR, (d) COX7B, (e) CAPG, (f) KCNN4, (g) MST1R, and (h) EMT score for paclitaxel resistance. [file 3534433.f3.pdf]

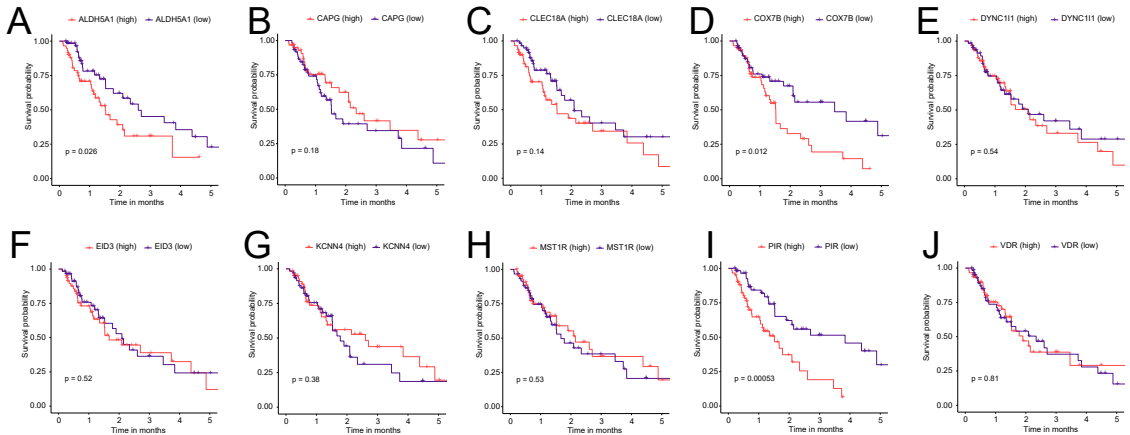

Supplement: Supplementary 4 — Supplementary Figure 4: Kaplan–Meier survival curves for genes for ALDH5A1 (a), CAPG (b), CLEC18A (c), COX7B (d), DYNC1I1 (e), EID3 (f), KCNN4 (g), MST1R (h), PIR (i), and VDR (j). [file 3534433.f4.pdf]
