# Supplementary material for: Epithelial-Mesenchymal Transition Gene Signature Is Associated with Neoadjuvant Chemoradiotherapy Resistance and Prognosis of Esophageal Squamous Cell Carcinoma
Source: Dis Markers. 2022 Aug 27;2022:3534433. doi: 10.1155/2022/3534433 (PMC9442501; doi:10.1155/2022/3534433)
Supplement: Supplementary 5 — Supplementary Table 1: the detailed information of samples from the GSE45670 dataset. Supplementary Table 2: the detailed information of samples from the TCGA-ESCC dataset. [file 3534433.f5.docx]

| Sample | Age | Gender | pathological response | Stage |
| --- | --- | --- | --- | --- |
| GSM1111672 | 61 | male | not pathological complete response | T3N1M0 |
| GSM1111673 | 43 | male | not pathological complete response | T3N1M0 |
| GSM1111674 | 59 | male | not pathological complete response | T3N1M0 |
| GSM1111675 | 50 | male | not pathological complete response | T3N1M0 |
| GSM1111676 | 59 | male | not pathological complete response | T3N1M0 |
| GSM1111677 | 55 | male | not pathological complete response | T3N1M0 |
| GSM1111678 | 54 | male | not pathological complete response | T2N1M0 |
| GSM1111679 | 53 | male | not pathological complete response | T3N1M0 |
| GSM1111680 | 57 | female | not pathological complete response | T2N1M0 |
| GSM1111681 | 53 | male | not pathological complete response | T2N1M0 |
| GSM1111682 | 60 | male | not pathological complete response | T2N1M0 |
| GSM1111683 | 54 | male | not pathological complete response | T3N1M0 |
| GSM1111684 | 54 | male | not pathological complete response | T3N1M0 |
| GSM1111685 | 69 | male | not pathological complete response | T3N1M0 |
| GSM1111686 | 53 | male | not pathological complete response | T3N1M0 |
| GSM1111687 | 53 | male | not pathological complete response | T3N1M0 |
| GSM1111688 | 59 | male | not pathological complete response | T3N1M0 |
| GSM1111689 | 62 | male | pathological complete response | T3N1M0 |
| GSM1111690 | 48 | male | pathological complete response | T3N1M0 |
| GSM1111691 | 56 | female | pathological complete response | T3N1M0 |
| GSM1111692 | 49 | male | pathological complete response | T2N1M0 |
| GSM1111693 | 59 | female | pathological complete response | T2N1M0 |
| GSM1111694 | 46 | male | pathological complete response | T3N1M0 |
| GSM1111695 | 64 | male | pathological complete response | T3N1M0 |
| GSM1111696 | 59 | male | pathological complete response | T3N1M0 |
| GSM1111697 | 65 | male | pathological complete response | T3N1M0 |
| GSM1111698 | 63 | male | pathological complete response | T2N1M0 |
| GSM1111699 | 61 | male | pathological complete response | T2N1M0 |

Supplementary Table 1. The detailed information of samples from the GSE45670 dataset.

| Barcode | Gender | OS | height | weight | OS.Time | Age | Stage |
| --- | --- | --- | --- | --- | --- | --- | --- |
| TCGA-2H-A9GF | MALE | 1 | 183 | 95 | 2.147945 | 67 | Stage III |
| TCGA-2H-A9GG | MALE | 1 | 178 | 74 | 1.671233 | 66 | Stage III |
| TCGA-2H-A9GH | MALE | 1 | 183 | 91 | 2.605479 | 44 | Stage IIB |
| TCGA-2H-A9GI | MALE | 1 | 188 | 100 | 1.191781 | 68 | Stage III |
| TCGA-2H-A9GJ | MALE | 1 | 189 | 70 | 4.879452 | 57 | Stage I |
| TCGA-2H-A9GK | MALE | 1 | 180 | 80 | 0.635616 | 43 | Stage III |
| TCGA-2H-A9GL | MALE | 1 | 173 | 85 | 0.493151 | 74 | Stage III |
| TCGA-2H-A9GM | MALE | 1 | 179 | 77 | 1.161644 | 53 | Stage IIB |
| TCGA-2H-A9GN | MALE | 1 | 185 | 82 | 0.745205 | 70 | Stage III |
| TCGA-2H-A9GO | MALE | 1 | 170 | 83 | 1.353425 | 58 | Stage IVA |
| TCGA-2H-A9GQ | MALE | 1 | 178 | 80 | 0.350685 | 80 | Stage III |
| TCGA-2H-A9GR | MALE | 1 | 168 | 67 | 2.70411 | 80 | Stage IIA |
| TCGA-IG-A5B8 | MALE | 1 | 170 | 64 | 0.065753 | 72 | Stage IB |
| TCGA-JY-A6FA | MALE | 1 | 174 | 64 | 3.728767 | 51 | Stage IIB |
| TCGA-JY-A6FE | MALE | 1 | 176 | 62 | 0.306849 | 49 | Stage III |
| TCGA-JY-A6FG | MALE | 1 | 173 | 110 | 3.460274 | 50 | Stage III |
| TCGA-L5-A43H | MALE | 1 | 175 | 69 | 0.024658 | 75 | Stage III |
| TCGA-L5-A43I | MALE | 1 | 172 | 121 | 1.523288 | 77 | Stage IIIA |
| TCGA-L5-A43J | MALE | 1 | 169 | 61 | 0 | 90 | Stage IIB |
| TCGA-L5-A4ON | MALE | 1 | 180 | 111 | 1.528767 | 65 | Stage IIB |
| TCGA-L5-A4OQ | MALE | 1 | 152 | 72 | 0.115068 | 75 | Stage IIIA |
| TCGA-L5-A4OR | MALE | 1 | 166 | 84 | 0.263014 | 83 | Stage IA |
| TCGA-L5-A4OT | MALE | 1 | 178 | 98 | 0.408219 | 77 | Stage IV |
| TCGA-L5-A4OW | FEMALE | 1 | 157 | 100 | 0.594521 | 56 | Stage IIB |
| TCGA-L5-A4OX | MALE | 1 | 183 | 110 | 0.619178 | 60 | Stage IIB |
| TCGA-L5-A88W | MALE | 1 | 182 | 80 | 2.093151 | 67 | Stage IIA |
| TCGA-L5-A8NF | MALE | 1 | 173 | 89 | 0.221918 | 57 | Stage IVA |
| TCGA-L5-A8NH | MALE | 1 | 183 | 96 | 1.076712 | 54 | Stage IV |
| TCGA-L5-A8NI | MALE | 1 | NA | 89 | 1.123288 | 79 | Stage III |
| TCGA-L5-A8NM | FEMALE | 1 | NA | 60 | 0.646575 | 84 | Stage IIB |
| TCGA-L5-A8NQ | MALE | 1 | 178 | 87 | 1.780822 | 71 | Stage IIA |
| TCGA-L5-A8NU | MALE | 1 | NA | 97 | 5.846575 | 84 | Stage IIA |
| TCGA-L5-A8NV | MALE | 1 | 183 | 82 | 4.380822 | 75 | Stage IIA |
| TCGA-L5-A8NW | MALE | 1 | 178 | 103 | 3.841096 | 55 |  |
| TCGA-Q9-A6FU | FEMALE | 1 | 158 | 59 | 0.430137 | 57 | Stage IIIB |
| TCGA-R6-A6DN | MALE | 1 | 175 | 98 | 0.665753 | 58 |  |
| TCGA-R6-A6DQ | FEMALE | 1 | 168 | 56 | 0.632877 | 74 |  |
| TCGA-R6-A6KZ | MALE | 1 | 180 | 71 | 0.421918 | 42 |  |
| TCGA-R6-A6L6 | MALE | 1 | 170 | 84 | 0.586301 | 68 |  |
| TCGA-R6-A6XQ | MALE | 1 | 178 | 85 | 0.528767 | 58 |  |
| TCGA-R6-A6Y2 | MALE | 1 | 169 | 89 | 0.775342 | 71 |  |
| TCGA-R6-A8W5 | MALE | 1 | 177 | 77 | 1.315068 | 60 | Stage IVA |
| TCGA-R6-A8W8 | MALE | 1 | 171 | 91 | 0.241096 | 72 |  |
| TCGA-R6-A8WG | MALE | 1 | 174 | 76 | 1.057534 | 60 |  |
| TCGA-RE-A7BO | FEMALE | 1 | 167 | 77 | 0.583562 | 72 | Stage IIB |
| TCGA-V5-A7RB | MALE | 1 | 172 | 133 | 0.441096 | 59 |  |
| TCGA-V5-A7RC | MALE | 1 | 171 | 76 | 0.284932 | 55 |  |
| TCGA-VR-A8EQ | MALE | 1 | 184 | 75 | 1.90137 | 73 | Stage III |
| TCGA-VR-A8ER | MALE | 1 | 164 | 46 | 1.035616 | 54 | Stage III |
| TCGA-VR-A8ET | MALE | 1 | 160 | 66 | 0.128767 | 64 | Stage IIA |
| TCGA-VR-A8EU | MALE | 1 | 174 | 51 | 1.526027 | 51 | Stage IV |
| TCGA-VR-A8EW | MALE | 1 | 164 | 48 | 0.676712 | 57 | Stage IIIB |
| TCGA-VR-A8EX | MALE | 1 | 171 | 60 | 2.342466 | 63 | Stage IVA |
| TCGA-VR-A8EZ | MALE | 1 | 167 | 69 | 1.515068 | 47 | Stage IIIC |
| TCGA-VR-AA7D | MALE | 1 | 166 | 57 | 0.764384 | 58 | Stage IIIC |
| TCGA-VR-AA7I | MALE | 1 | 171 | 56 | 1.326027 | 70 | Stage III |
| TCGA-XP-A8T6 | MALE | 1 | NA | 70 | 2.090411 | 54 | Stage IIB |
| TCGA-IC-A6RE | MALE | 0 | 183 | 102 | 0.641096 | 59 | Stage IIB |
| TCGA-IC-A6RF | FEMALE | 0 | 170 | 100 | 0.517808 | 69 | Stage IA |
| TCGA-IG-A3I8 | FEMALE | 0 | 165 | 48 | 0 | 51 | Stage IIA |
| TCGA-IG-A3QL | MALE | 0 | 170 | 73 | 0 | 54 | Stage IIA |
| TCGA-IG-A3Y9 | MALE | 0 | 176 | 80 | 0 | 72 | Stage IIIA |
| TCGA-IG-A3YA | MALE | 0 | 179 | 76 | 0 | 53 | Stage IIIA |
| TCGA-IG-A3YB | MALE | 0 | 178 | 80 | 0 | 61 | Stage IIIA |
| TCGA-IG-A3YC | MALE | 0 | 169 | 66 | 0 | 62 | Stage IIIA |
| TCGA-IG-A4P3 | MALE | 0 | 182 | 86 | 0.00274 | 48 | Stage IIB |
| TCGA-IG-A4QS | MALE | 0 | 172 | 78 | 0.021918 | 71 | Stage IIIB |
| TCGA-IG-A4QT | MALE | 0 | 174 | 70 | 0.082192 | 56 | Stage IIA |
| TCGA-IG-A50L | MALE | 0 | 162 | 61 | 0.043836 | 58 | Stage IIIA |
| TCGA-IG-A51D | MALE | 0 | 178 | 63 | 0 | 63 | Stage IIB |
| TCGA-IG-A5S3 | FEMALE | 0 | 179 | 69 | 0 | 69 | Stage IIB |
| TCGA-IG-A625 | MALE | 0 | 168 | 50 | 0.030137 | 60 | Stage IIIB |
| TCGA-IG-A6QS | MALE | 0 | 176 | 70 | 0.010959 | 54 | Stage IIB |
| TCGA-IG-A7DP | FEMALE | 0 | 166 | 58 | 0.093151 | 50 | Stage IIIA |
| TCGA-IG-A8O2 | MALE | 0 | 166 | 53 | 0.038356 | 62 | Stage IIIB |
| TCGA-IG-A97H | MALE | 0 | 170 | 59 | 0.035616 | 36 | Stage IIA |
| TCGA-IG-A97I | MALE | 0 | 174 | 62 | -0.01096 | 58 | Stage IIA |
| TCGA-JY-A6F8 | MALE | 0 | 188 | 90 | 10.17534 | 56 | Stage I |
| TCGA-JY-A6FB | MALE | 0 | 177 | 95 | 5.032877 | 77 | Stage I |
| TCGA-JY-A6FD | FEMALE | 0 | 152 | 65 | 4.487671 | 51 | Stage IIA |
| TCGA-JY-A6FH | MALE | 0 | 180 | 95 | 3.008219 | 53 | Stage IIB |
| TCGA-JY-A938 | MALE | 0 | 165 | 72 | 2.117808 | 75 | Stage IIB |
| TCGA-JY-A939 | MALE | 0 | 180 | 89 | 1.290411 | 77 | Stage IIB |
| TCGA-JY-A93C | MALE | 0 | 169 | 62 | 1.931507 | 47 | Stage IIIB |
| TCGA-JY-A93D | MALE | 0 | 197 | 52 | 2.106849 | 51 | Stage IIIC |
| TCGA-JY-A93E | MALE | 0 | 173 | 73 | 1.506849 | 61 | Stage IIIA |
| TCGA-JY-A93F | FEMALE | 0 | 165 | 73 | 1.465753 | 58 | Stage IB |
| TCGA-KH-A6WC | MALE | 0 | 165 | 72 | 0.517808 | 82 | Stage IA |
| TCGA-L5-A43C | MALE | 0 | 155 | 95 | 0.263014 | 81 |  |
| TCGA-L5-A43E | MALE | 0 | 183 | 74 | 0.287671 | 74 | Stage I |
| TCGA-L5-A43M | FEMALE | 0 | 164 | 89 | 0.745205 | 84 |  |
| TCGA-L5-A4OE | MALE | 0 | NA | 88 | 0.293151 | 81 | Stage IIIB |
| TCGA-L5-A4OF | MALE | 0 | 179 | 193 | 0.306849 | 63 | Stage IIB |
| TCGA-L5-A4OG | FEMALE | 0 | 163 | 67 | 0.279452 | 79 | Stage I |
| TCGA-L5-A4OH | MALE | 0 | 175 | 127 | 1.624658 | 71 | Stage I |
| TCGA-L5-A4OI | MALE | 0 | 173 | 83 | 0.731507 | 79 | Stage IIIC |
| TCGA-L5-A4OJ | FEMALE | 0 | 164 | 114 | 0.534247 | 70 | Stage I |
| TCGA-L5-A4OM | FEMALE | 0 | NA | 69 | 0.339726 | 54 | Stage IA |
| TCGA-L5-A4OO | MALE | 0 | 178 | 77 | 0.276712 | 75 | Stage IIIC |
| TCGA-L5-A4OP | FEMALE | 0 | 150 | 71 | 0.59726 | 67 | Stage IA |
| TCGA-L5-A4OS | FEMALE | 0 | 157 | 59 | 2.561644 | 86 | Stage IIB |
| TCGA-L5-A4OU | MALE | 0 | 175 | 82 | 2.416438 | 81 | Stage IIA |
| TCGA-L5-A88S | MALE | 0 | 183 | 77 | 0.578082 | 84 | Stage IB |
| TCGA-L5-A88T | MALE | 0 | 174 | 80 | 0.726027 | 86 | Stage IIB |
| TCGA-L5-A88V | MALE | 0 | 175 | 73 | 0.216438 | 60 | Stage III |
| TCGA-L5-A88Y | MALE | 0 | 185 | 76 | 0.030137 | 76 |  |
| TCGA-L5-A88Z | FEMALE | 0 | 163 | 66 | 0.616438 | 70 | Stage IIA |
| TCGA-L5-A891 | MALE | 0 | 183 | 81 | 0.312329 | 51 |  |
| TCGA-L5-A893 | FEMALE | 0 | 150 | 67 | 0.252055 | 71 | Stage I |
| TCGA-L5-A8NE | MALE | 0 | 170 | 94 | 4.624658 | 77 | Stage IIB |
| TCGA-L5-A8NG | MALE | 0 | 166 | 81 | 2.99726 | 77 | Stage III |
| TCGA-L5-A8NJ | MALE | 0 | 173 | 81 | 1.372603 | 77 | Stage III |
| TCGA-L5-A8NK | FEMALE | 0 | 161 | 67 | 1.128767 | 84 | Stage IIA |
| TCGA-L5-A8NL | MALE | 0 | 158 | 80 | 1.10137 | 56 | Stage III |
| TCGA-L5-A8NN | MALE | 0 | 170 | 60 | 0.452055 | 81 | Stage III |
| TCGA-L5-A8NR | FEMALE | 0 | 157 | 60 | 0.726027 | 81 | Stage III |
| TCGA-L5-A8NS | MALE | 0 | 180 | 88 | 1.117808 | 76 | Stage IIB |
| TCGA-L5-A8NT | MALE | 0 | 183 | 84 | 2.260274 | 69 | Stage IIB |
| TCGA-L7-A56G | MALE | 0 | 170 | 91 | 0.671233 | 65 |  |
| TCGA-L7-A6VZ | MALE | 0 | 185 | 111 | 0.728767 | 62 | Stage IIIC |
| TCGA-LN-A49K | MALE | 0 | 160 | 57 | 0.005479 | 66 | Stage IIA |
| TCGA-LN-A49L | MALE | 0 | 167 | 62 | 0.005479 | 44 | Stage IIA |
| TCGA-LN-A49M | MALE | 0 | 168 | 57 | 0.013699 | 62 | Stage IIA |
| TCGA-LN-A49N | MALE | 0 | 168 | 54 | 0.005479 | 50 | Stage IIB |
| TCGA-LN-A49O | MALE | 0 | 158 | 57 | 0.027397 | 47 | Stage IIA |
| TCGA-LN-A49P | MALE | 0 | 175 | 62 | 0 | 71 | Stage IIA |
| TCGA-LN-A49R | MALE | 0 | 175 | 62 | 0.008219 | 46 | Stage III |
| TCGA-LN-A49S | MALE | 0 | 173 | 63 | 0.010959 | 59 | Stage IIA |
| TCGA-LN-A49U | MALE | 0 | 178 | 64 | 0.005479 | 62 | Stage IIA |
| TCGA-LN-A49V | MALE | 0 | 173 | 62 | 0.008219 | 49 | Stage IIA |
| TCGA-LN-A49W | MALE | 0 | 162 | 61 | 0.005479 | 73 | Stage III |
| TCGA-LN-A49X | MALE | 0 | NA | NA | 0.008219 | 44 | Stage IIA |
| TCGA-LN-A49Y | MALE | 0 | 168 | 57 | 0.005479 | 77 | Stage IIA |
| TCGA-LN-A4A1 | MALE | 0 | 172 | 65 | 0 | 60 | Stage IIA |
| TCGA-LN-A4A2 | MALE | 0 | 165 | 58 | 0.010959 | 57 | Stage IIA |
| TCGA-LN-A4A3 | MALE | 0 | 172 | 63 | 0.005479 | 61 | Stage III |
| TCGA-LN-A4A4 | MALE | 0 | 175 | 62 | 0.008219 | 36 | Stage III |
| TCGA-LN-A4A5 | MALE | 0 | 175 | 68 | 0.008219 | 49 | Stage IIA |
| TCGA-LN-A4A6 | MALE | 0 | 176 | 72 | 0.008219 | 65 | Stage II |
| TCGA-LN-A4A8 | MALE | 0 | 175 | 68 | 0.005479 | 52 | Stage IIA |
| TCGA-LN-A4A9 | MALE | 0 | 178 | 72 | 0.005479 | 58 | Stage IIA |
| TCGA-LN-A4MQ | MALE | 0 | 168 | 56 | 0.008219 | 46 | Stage III |
| TCGA-LN-A4MR | MALE | 0 | 162 | 59 | 0.005479 | 57 | Stage IIA |
| TCGA-LN-A5U5 | MALE | 0 | 164 | 47 | 0.013699 | 57 | Stage IV |
| TCGA-LN-A5U6 | MALE | 0 | 172 | 60 | 0.016438 | 54 | Stage IIB |
| TCGA-LN-A5U7 | MALE | 0 | 169 | 53 | 0.09863 | 46 | Stage IIA |
| TCGA-LN-A7HV | MALE | 0 | 174 | 72 | 0 | 58 | Stage IIA |
| TCGA-LN-A7HW | MALE | 0 | 176 | 69 | 0 | 59 | Stage IIA |
| TCGA-LN-A7HX | MALE | 0 | 172 | 67 | 0 | 72 | Stage IIA |
| TCGA-LN-A7HY | MALE | 0 | 176 | 65 | 0 | 50 | Stage III |
| TCGA-LN-A7HZ | MALE | 0 | 176 | 62 | 0 | 49 | Stage IIA |
| TCGA-LN-A8HZ | MALE | 0 | 175 | 63 | 0 | 56 | Stage IIA |
| TCGA-LN-A8I0 | MALE | 0 | 171 | 65 | 0 | 52 | Stage IIA |
| TCGA-LN-A8I1 | FEMALE | 0 | 152 | 61 | 0 | 67 | Stage IIA |
| TCGA-LN-A9FO | MALE | 0 | 170 | 65 | 0.010959 | 42 | Stage IIA |
| TCGA-LN-A9FP | FEMALE | 0 | 151 | 49 | 0 | 60 | Stage IIA |
| TCGA-LN-A9FQ | MALE | 0 | 172 | 63 | 0 | 62 | Stage IIA |
| TCGA-LN-A9FR | MALE | 0 | 172 | 61 | 0 | 70 | Stage IIB |
| TCGA-M9-A5M8 | MALE | 0 | 174 | 51 | 1.468493 | 58 | Stage IIA |
| TCGA-Q9-A6FW | MALE | 0 | 170 | 79 | 0.315068 | 61 | Stage IIIB |
| TCGA-R6-A6L4 | MALE | 0 | 179 | 106 | 1.224658 | 27 |  |
| TCGA-R6-A6XG | MALE | 0 | 192 | 103 | 2.465753 | 64 |  |
| TCGA-R6-A6Y0 | MALE | 0 | 173 | 94 | 4.49589 | 54 |  |
| TCGA-R6-A8WC | MALE | 0 | 180 | 89 | 0.060274 | 56 |  |
| TCGA-S8-A6BV | MALE | 0 | 180 | 90 | 0.731507 | 76 | Stage IIIA |
| TCGA-S8-A6BW | MALE | 0 | 177 | 76 | 1.167123 | 51 | Stage IB |
| TCGA-V5-A7RE | MALE | 0 | 169 | 77 | 0.4 | 45 | Stage IB |
| TCGA-V5-AASV | MALE | 0 | 165 | 55 | 0.706849 | 67 | Stage IIB |
| TCGA-V5-AASW | MALE | 0 | 175 | 88 | 0.679452 | 72 |  |
| TCGA-V5-AASX | MALE | 0 | 192 | 100 | 0.369863 | 74 |  |
| TCGA-VR-A8EO | MALE | 0 | 179 | 63 | 1.394521 | 49 | Stage IIA |
| TCGA-VR-A8EP | MALE | 0 | 173 | 59 | 1.29863 | 51 | Stage IIIB |
| TCGA-VR-A8EY | FEMALE | 0 | 165 | 70 | 1.983562 | 44 | Stage IIA |
| TCGA-VR-A8Q7 | MALE | 0 | 170 | 57 | 3.060274 | 60 | Stage IIIA |
| TCGA-VR-AA4D | MALE | 0 | 178 | 82 | 3.775342 | 53 | Stage IIB |
| TCGA-VR-AA4G | FEMALE | 0 | 159 | 69 | 1.00274 | 51 | Stage IIIA |
| TCGA-VR-AA7B | FEMALE | 0 | 159 | 47 | 0.936986 | 65 | Stage IV |
| TCGA-X8-AAAR | MALE | 0 | 183 | 105 | 0.846575 | 69 |  |
| TCGA-XP-A8T7 | FEMALE | 0 | NA | 51 | 3.435616 | 63 | Stage IIA |
| TCGA-XP-A8T8 | MALE | 0 | NA | 62 | 1.19726 | 49 | Stage IIB |
| TCGA-Z6-A8JD | MALE | 0 | 172 | 69 | 0.054795 | 53 | Stage IIB |
| TCGA-Z6-A8JE | MALE | 0 | 168 | 61 | 0 | 57 | Stage IIIA |
| TCGA-Z6-A9VB | MALE | 0 | 159 | 62 | 0.041096 | 53 | Stage IIIA |
| TCGA-Z6-AAPN | MALE | 0 | 165 | 74 | 0.049315 | 57 | Stage IIA |
| TCGA-ZR-A9CJ | MALE | 0 | NA | NA | 1.509589 | 65 | Stage IIIC |

Supplementary Table 2. The detailed information of samples from the TCGA-ESCC dataset.
